# Supplementary material for: Biotransformation of chlorothalonil by strain Stenotrophomonas acidaminiphila BJ1 isolated from farmland soil
Source: R Soc Open Sci. 2019 Nov 6;6(11):190562. doi: 10.1098/rsos.190562 (PMC6894561; doi:10.1098/rsos.190562)
Supplement: Data of degradation and chromatogram of chlorothalonil degradation [file rsos190562supp1.docx]

Electronic sumplementary materials for “Biotransformation of chlorothalonil by strain Stenotrophomonas acidaminiphila BJ1 isolated from farmland soil”

**Qingming Zhang^*1^, Hongyu, Liu^1^, Muhammad Saleem^2^ Caixia Wang^1^**

*^1^Key Lab of Integrated Crop Pest Management of Shandong Province, College of Plant Health and Medicine, Qingdao Agricultural University, Qingdao, 266109, China*

*^2^Department of Biological Sciences, Alabama State University, Montgomery, AL 36101, USA*


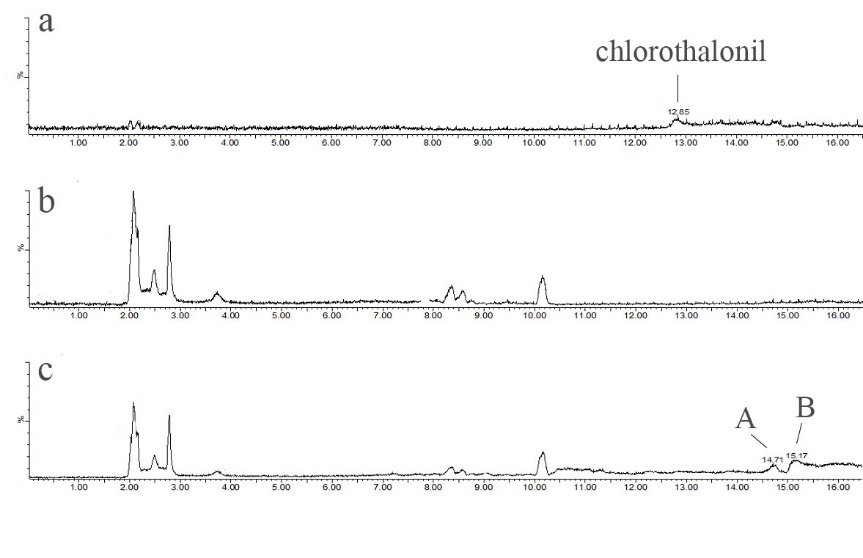


**Figure S1** HPLC elution curves of chlorothalonil and metabolites. a: chlorothalonil standard; b: strain BJ1 in MSM; c: chlorothalonil and strain BJ1 in MSM.
